# Heatstroke presentations to urban hospitals during BC’s extreme heat event: lessons for the future

**DOI:** 10.1007/s43678-023-00622-y

**Published:** 2023-12-28

**Authors:** Kira Gossack-Keenan, David Seonguk Yeom, Josephine Kanu, Jeffrey P. Hau, Rhonda J. Rosychuk, Dylan Clark, Rajan Bola, Caris Tze, Chris Niosco, Hayley Emery, Phillip Yeung, Corinne M. Hohl

**Affiliations:** 1 Department of Emergency Medicine, University of British Columbia, Vancouver, BC Canada; 2 Centre for Clinical Epidemiology and Evaluation, Vancouver Coastal Health Research Institute, Vancouver, BC Canada; 3 Department of Pediatrics, University of Alberta, Edmonton, Canada; 4 Climate Institute Canada, Vancouver, BC Canada; 5 Emergency Department, Vancouver General Hospital, Vancouver, BC Canada; 6 Faculty of Medicine, Diamond Health Care Centre, 11th Floor, 2775 Laurel Street, Vancouver, BC V5Z 1M9 Canada

**Keywords:** Heastroke, Heatwave, Climate, Emergency medicine, Emergency department

## Abstract

**Background:**

Climate change is leading to more extreme heat events in temperate climates that typically have low levels of preparedness. Our objective was to describe the characteristics, treatments, and outcomes of adults presenting to hospitals with heatstroke during BC’s 2021 heat dome.

**Methods:**

We conducted a review of consecutive adults presenting to 7 hospitals in BC’s Lower Mainland. We screened the triage records of all patients presenting between June 25th and 30th, 2021 for complaints related to heat, and reviewed the full records of those who met heatstroke criteria. Our primary outcome was in-hospital mortality. We used Mann–Whitney *U* tests and logistic regression to investigate associations between patient and treatment factors and mortality.

**Results:**

Among 10,247 consecutive presentations to urban hospitals during the extreme heat event, 1.3% (139; 95% confidence intervals [CI] 1.1–1.6%) met criteria for heatstroke. Of heatstroke patients, 129 (90.6%) were triaged into the two highest acuity levels. Patients with heatstroke had a median age of 84.4 years, with 122 (87.8%) living alone, and 101 (84.2%) unable to activate 911 themselves. A minority (< 5, < 3.6%) of patients presented within 48 h of the onset of extreme heat. Most patients (107, 77.0%) required admission, and 11.5% (16) died in hospital. Hypotension on presentation was associated with mortality (odds ratio [OR] 5.3).

**Interpretation:**

Heatstroke patients were unable to activate 911 themselves, and most presented with a 48-h delay. This delay may represent a critical window of opportunity for pre-hospital and hospital systems to prepare for the influx of high-acuity resource-intensive patients.

**Supplementary Information:**

The online version contains supplementary material available at 10.1007/s43678-023-00622-y.

## Résumé

**Contexte:**

Les changements climatiques entraînent une augmentation des épisodes de chaleur extrême dans les climats tempérés qui ont généralement de faibles niveaux de préparation. Notre objectif était de décrire les caractéristiques, les traitements et les résultats des adultes présentant un coup de chaleur à l’hôpital pendant le dôme de chaleur de 2021 en Colombie-Britannique.

**Méthodes:**

Nous avons effectué un examen des adultes consécutifs qui se sont présentés dans sept hôpitaux du Lower Mainland de la Colombie-Britannique. Nous avons examiné les dossiers de triage de tous les patients qui se sont présentés entre le 25 et le 30 juin 2021 pour les plaintes liées à la chaleur et examiné les dossiers complets de ceux qui répondaient aux critères de coup de chaleur. Notre principal résultat était la mortalité à l’hôpital. Nous avons utilisé les tests de Mann-Whitney U et la régression logistique pour étudier les associations entre le patient et les facteurs de traitement et la mortalité.

**Résultats:**

Parmi les 10247 présentations consécutives aux hôpitaux urbains pendant l’événement de chaleur extrême, 1,3 % (139; intervalles de confiance [IC] à 95 %) répondaient aux critères de coup de chaleur. Parmi les patients ayant subi un coup de chaleur, 129 (90,6 %) ont été classés dans les deux niveaux d’acuité les plus élevés. Les patients atteints d’un coup de chaleur avaient un âge médian de 84,4 ans, 122 (87,8 %) vivant seuls et 101 (84,2 %) incapables d’activer le 911 eux-mêmes. Une minorité (< 5, < 3,6 %) de patients se sont présentés dans les 48 heures suivant l’apparition de la chaleur extrême. La plupart des patients (107, 77,0 %) ont dû être admis et 11,5 % (16) sont décédés à l’hôpital. L’hypotension au moment de la présentation était associée à la mortalité (rapport de cotes [RC] 5.3).

**Interprétation:**

Les patients atteints d’un coup de chaleur n’ont pas pu activer le 911 eux-mêmes, et la plupart se sont présentés avec un délai de 48 heures. Ce délai peut représenter une fenêtre critique d’opportunité pour les systèmes préhospitaliers et hospitaliers de se préparer à l’afflux de patients à forte intensité de ressources.

## Clinician’s Capsule

**Table:** 

***What is known about the topic?***
Emergency departments are likely to see more heatstroke cases in temperate areas with low levels of preparedness.
***What did this study ask?***
What are the characteristics, treatments, and outcomes of heatstroke patients who survived to hospital in a region that historically has had a temperate climate?
***What did this study find?***
Heatstroke patients were elderly, unable to activate 911 themselves, and presented after 48-h. Simple measures achieved rapid cooling, yet one in 8 patients died in hospital.
***Why does this study matter to clinicians?***
Emergency Departments have 48 h to prepare for a rapid influx of high-acuity patients who will require substantial resources.

## Introduction

Climate change is leading to more frequent and severe extreme heat events [1]. Until recently, heat-related illness was more common in warmer climates. However, temperate areas are increasingly experiencing periods of extreme heat, which have devastating impacts on population-level mortality [2]. In 2021, BC experienced an exceptional heat wave that brought record temperatures into a geographic area with a temperate climate with very limited population and system-level preparedness [12]. Emergency Medical Services (EMS) and hospitals in BC’s Lower Mainland were massively overwhelmed, and population mortality quadrupled during this time [15]. There is an urgent need to understand heat-related illness and its impact on health services use during extreme heat events in temperate areas with low baseline levels of population and system-level preparedness, so that evidence-based mitigation strategies can be developed, evaluated, and implemented.

Heat-related illness occurs when the body’s thermoregulatory responses are overwhelmed and unable to preserve homeostasis. Heatstroke is a medical emergency, which results in multiorgan failure and death [3–5]. During heatwaves, populations living in temperate areas are more prone to heat-related illness including heatstroke, because they lack physiologic acclimation to extreme heat, which requires repeated exposures to heat [6, 7]. This is compounded by low levels of population and system-level preparedness, as extreme heat events have thus far been rare [8]. The most notable example of how deadly inadequate preparation for extreme heat can be was France’s 2003 heat wave, which caused nearly 15,000 deaths [9–11].

Our goal was to describe patients with heatstroke who presented to urban hospitals in BC’s Lower Mainland during the extreme heat event to inform the development of prevention and mitigation strategies. Our objectives were to examine the characteristics, treatments, and outcomes of heatstroke patients who survived to hospital, and identify factors associated with mortality.

## Methods

### Study design and setting

We conducted a multi-centre chart review of consecutive adults presenting with heatstroke to seven BC hospitals. We included hospitals across Vancouver and Surrey, and their surrounding metropolitan areas that were part of the Canadian COVID-19 Emergency Department Rapid Response Network (CCEDRRN) [16–20]. These consisted of three urban tertiary care teaching hospitals (Vancouver General Hospital, Saint Paul’s Hospital, and Royal Columbian Hospital) and four urban community hospitals (Mount Saint Joseph’s Hospital, Lion’s Gate Hospital, Surrey Memorial Hospital, Eagle Ridge Hospital; Appendix Table 1). None of these hospitals had defined treatment protocols for heat-related illnesses at the time of the extreme heat event. The University of British Columbia Clinical Research Ethics Board approved the study (H21-02763) and waived the need for informed consent.

### Patients

We included patients diagnosed with heatstroke who presented to a participating ED between June 25th and 30th, 2021. We excluded patients less than 16 years of age or diagnosed with an alternative diagnosis that explained their presentation (e.g., sepsis).

### Study period

We defined the study period based the most common definition for heat wave: 3 or more consecutive days with daily maximum (Tmax) and minimum (Tmin) temperatures greater than the historical 90th percentile, based on temperatures recordings at Vancouver International Airport compared to a 30-year baseline (years 1991–2020; Appendix Table 2) [21, 22]. The heatwave was between June 25 and 29th, 2021. We included June 30th to capture patients who presented with residual effects of heat exposure.

### Data collection

CCEDRRN research assistants were blinded to the study protocol and objectives. They screened the triage documentation of consecutive ED patients who presented during the study period for a list of complaints compatible with heatstroke (Appendix Table 3) [23]. When patients met screening criteria, research assistants reviewed pre-hospital, ED, and inpatient medical records to determine whether they presented with heatstroke. A physician subsequently reviewed all records with heatstroke presentations to ensure that all alternative diagnoses had been identified. Research assistants collected data on patient demographics, clinical data (vital signs, comorbidities, the Canadian Triage and Acuity Scale (CTAS) reflecting the patient’s acuity, whereby CTAS 1 indicates the highest acuity level), laboratory data, pre-hospital and hospital treatments, and patient outcomes (time to hospital presentation, admission, and in-hospital mortality) [24].

### Definitions

We defined heatstroke as initial presentation with hyperthermia (any documented body temperature of ≥ 39 °C) in addition to delirium, seizures, altered level of consciousness, or a Glasgow Coma Scale (GCS) of ≤ 8 in the absence of an alternative cause on initial presentation [4]. Initial presentation was based on documentation in EHS and/or ED records. We used a lower temperature cut-off as recommended by Bouchama et al., because many documented pre-hospital and ED temperatures were obtained peripherally which are estimated to be 1–1.5 °C lower than core temperatures [25]. This allowed us to include hyperthermic patients who had been cooled by bystanders and pre-hospital personnel before a core temperature could be obtained [26].

### Outcomes

Our primary outcome was any in-hospital mortality in the ED or during hospital admission. We categorized patients who were discharged from hospital as alive. Secondary outcomes were the delay from the onset of the extreme heat to hospital presentation and hospital admission.

### Statistical analysis

We used descriptive statistics to summarize the characteristics and treatments of heatstroke patients, stratified by survival status at hospital discharge. We assessed distribution of continuous variables by Shapiro–Wilk test and described them using either mean and standard deviation (SD), or median and interquartile range (IQR). We described categorical variables using frequencies and percentages. We used the Mann–Whitney *U* test to compare laboratory data by survivor status. We presented univariable logistic regression analyses. The limited number of in-hospital mortality events did not permit multivariable modeling. We considered a p value of less than 0.05 statistically significant. We completed analyses in R version 4.1.2 [27].

## Results

**Fig. 1:**
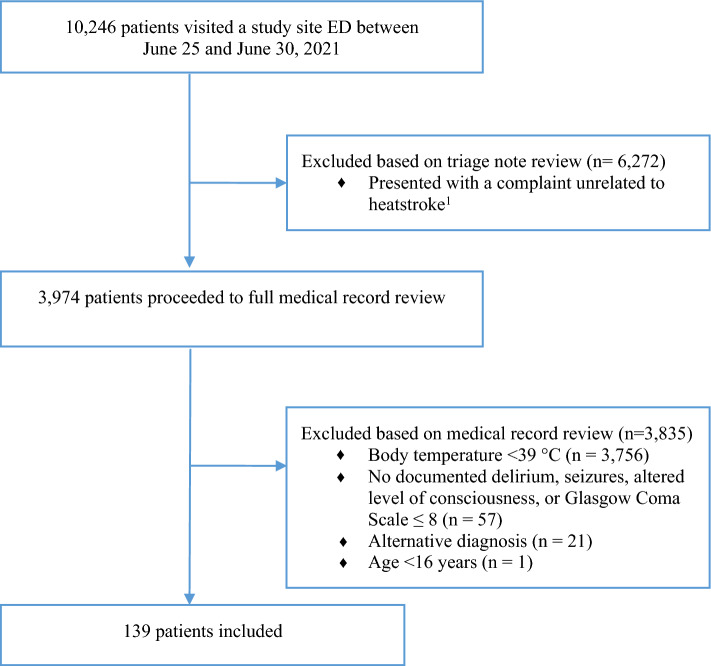
Patient flow diagram. *ED* emergency department. ^1^Eligible presenting complaints included heat-related illness, altered level of consciousness, general weakness, confusion, seizure, shortness of breath, fever, dehydration, headache, nausea or vomiting, bizarre behaviour, palpitations/irregular heartbeat, syncope/pre-syncope, cardiac arrest (non-traumatic), chest pain, vertigo, gait disturbance/ataxia, extremity weakness/symptoms of cerebrovascular accident, sensory loss/parasthesias, or hypoglycemia

Among 10,247 consecutive patients who presented to a participating ED during the extreme heat event, 139 (1.3%, 95% CI 1.1–1.6%) were diagnosed with heatstroke (Fig. 1). Their median age was 84.4 years (Table 1). Most were female (54.6%), lived alone in a private residence (87.8%), and had a 911 call placed by someone else (84.2%). Most patients were triaged as CTAS category 1 (45.3%) requiring immediate physician attention, or CTAS category 2 (45.3%) requiring physician assessment within 15 min of arrival, highlighting their extremely high acuity. The most common presenting complaint was altered level of consciousness (56.1%). Common comorbidities were hypertension (56.8%), diabetes (32.4%), and psychiatric conditions (26.6%). Most patients were severely hyperthermic (median temperature 39.6 °C).

**Table 1:** Characteristics of heatstroke patients stratified by survival to hospital discharge*

Demographics	All patients (*n* = 139)	Survivors (*n* = 123)	Non-survivors (n = 16)	*p* value
Age in years, (median)	84.4	84.0	86.9	0.27
Female, (%)	54.6	55.4	56.2	0.95
Place of residence, (%)				
Home	87.8	86.9	100	0.50
Long-term care/rehab facility	6.5	7.4	0	
No fixed address/shelter/single room occupancy	3.6	4.1	0	
Retirement home	< 5^1^	< 5	0	
Arrival mode, (%)				
Self	12.2	12.2	< 5	0.87
EMS/police	87.8	87.8	87.5	
EMS contact initiation, (%)**				
Initiated by anyone other than self	84.2	83.0	92.9	0.81
Initiated by self/unclear	15.8	17.0	< 5	
CTAS score, (%)				
1 (Resuscitation)	45.3	39.8	87.5	< 0.01
2 (Emergent)	45.3	49.6	< 5	
3 (Urgent)	6.5	7.3	0	
4 (Less urgent)	< 5	< 5	0	
5 (Non-urgent)	0	0	0	
EMS call to ED arrival, minutes (median)	69.0	69.5	50.0	< 0.01
Initial vital signs				
Heart rate, beats per min (mean)	114.2	115.0	108.3	0.54
Systolic blood pressure, mm Hg (mean)	132.4	134.3	116.7	0.14
Oxygen saturation, % (median)	92.0	91.0	96.5	0.79
Respiratory rate, breaths per min (median)	30.0	30.0	28.0	0.37
Temperature, degree Celsius (median)	39.6	39.9	39.8	0.88
Glasgow Coma Scale (median)	10.5	11.0	4.0	< 0.01
Most common comorbid conditions, (%)				
Hypertension	56.8	56.9	56.3	0.99
Diabetes	32.4	33.3	< 5	0.70
Psychiatric condition/mental health diagnosis	26.7	28.5	< 5	0.29
Congestive heart failure	20.1	18.7	31.3	0.40
Chronic kidney disease	13.7	13.8	< 5	0.99
Coronary artery disease	13.7	11.4	31.3	0.26
Chronic obstructive pulmonary disease	11.5	13.0	0	0.26
Dementia	11.5	11.4	< 5	0.99
Cerebrovascular disease	5.8	6.5	0	0.63
Most common triage presenting complaints, (%)				
Altered level of consciousness	56.1	56.9	50.0	0.80
Heat-related illness	13.7	15.4	0	0.19
General weakness	5.0	4.9	< 5	0.99
Confusion	3.6	4.1	0	0.91
Syncope/pre-syncope cardiac arrest	3.6	4.1	0	0.91
Fever	3.6	< 5	< 5	0.99
Seizure	3.6	< 5	< 5	0.19
Extremity weakness/stroke symptoms		< 5	0	0.99
Other***	6.5	4.1	< 5	< 0.01

*IQR* interquartile range, *EMS* Emergency Medical System, *CTAS* Canadian Triage Acuity Scale, *ED* emergency department

*To ensure patient privacy, a cell size restriction policy prohibited reporting counts of less than 5

**The denominators for EMS contact initiation are 120 for all patients, 106 for survived to hospital discharge, and 14 for died in hospital

***Other complaints included respiratory distress, cardiac arrest, and respiratory arrest

Most patients (95.7%) presented on days 3–5 of the heat wave (Appendix Fig. 2a). The majority (91.4%) were actively cooled (Appendix Table 4). Among heatstroke patients, 63.3% achieved a body temperature of < 38 °C within 180 min (Appendix Fig. 1). Nineteen-point four percent of patients required mechanical ventilation (Appendix Table 4). The median ED length of stay (LOS) for all heatstroke patients was 8.2 h (Appendix Table 5). In the ED, 18% of patients were discharged and five patients died (3.6%). Seventy-seven percent were admitted, 58.3% to a ward and 13.7% to intensive care. The median in-hospital LOS was 9.0 days. Seven-point nine percent died after admission (overall in-hospital mortality 11.5%). Non-survivors had more abnormal laboratory values reflecting a greater degree of shock (Appendix Table 6). In univariate analyses, hypotension increased the odds of death 5.3-fold (Appendix Table 7).

## Discussion

### Interpretation

At a time when climate science is irrefutably pointing towards increased frequency and severity of extreme weather events, health systems in temperate climates must prepare for heat-related illness [28]. Our multi-centre study of BC’s 2021 extreme heat event found that most patients presenting to hospital with heatstroke lived alone in private residences and were unable to activate 911 themselves. The vast majority of patients had a delay of over 48 h into the heat event before they presented to hospital. This represents an important window for EMS systems, EDs, and hospitals to prepare for an influx of high-acuity patients. Even though only 1.4% of patients who presented to hospitals during the extreme weather event had heatstroke, they had extremely high acuity. Despite achieving rapid cooling in most patients, one in eight patients who was transported alive to hospital died, many within 72 h of arrival. Interestingly, our findings are consistent with BC Coroner data which demonstrated that most deaths occurred in older adults with underlying comorbidities who lived alone, with most 911 calls placed after the third day of the heat wave. Most deaths were not reported until after the third day of the extreme heat [14].

### Previous studies

Only one large US administrative database study reported lower mortality than our study but did not focus on geographically temperate areas with low levels of population and system-level preparedness. Much of the US lies in warm to hot semi-arid and dessert regions where physiologic acclimation and rates of indoor cooling are greater than in Canada (90% versus 60% of the population with air conditioner access) [29, 30]. In addition, the authors relied on administrative data to identify heatstroke patients, which may have resulted in ascertainment bias, and may have led to inclusion of exertional heatstroke patients. The high in-hospital mortality we observed compared favorably with the previous reports of 5–50% mortality, despite BC’s low population and system-level preparedness [3, 10, 11, 31, 32]. Compared to other geographic areas, we found more patients in our study received cooling (91% compared to 56% during France’s 2003 heatwave with 62% mortality [9–11].

### Strengths and limitations

Our study is the largest multisite clinical study on the characteristics, treatments, and outcomes of heatstroke patients across pre-hospital, ED, and hospital settings, and fills an important gap in the literature by focusing on heatstroke presentations in a region with a temperate climate. It provides critical information about the fluctuations in the number and timing of presentations over the heatwave course that can inform efforts to prepare for future events. We applied a pragmatic lens to our definition of heatstroke, as many patients in our cohort did not have timely measurement of core temperatures. Core temperatures are more difficult to obtain in the field than peripheral temperatures, in particular in patients with altered level of consciousness [15]. Based on the report to the Chief Coroner, the heat wave doubled 911 calls and placed extreme stress upon EMS [15]. This caused delays in call operator and EMS response times. Thus, cooling was often initiated by those who called 911 before a core temperature could be measured. Our pragmatic definition helped capture patients who likely met the traditional core temperature cut-off in the field. Our study is not without limitations. We captured data which resulted in information bias, as we were only able to capture what was documented in medical records. Similar to prior studies, we were only able to include patients who survived to hospital arrival, resulting in survivorship bias. Our results should therefore be applied to those patients who survive to hospital, and must be considered in the context of the BC Coroner’s report which summarized findings about those who died [15]. Finally, despite including seven sites located in the epicentre of the heatwave, relatively few patients met heatstroke criteria, limiting our ability to conduct multivariable analyses. This indicates that larger multisite studies on heatstroke are needed.

### Clinical implications

Interestingly, the vast majority of patients did not present to hospital until the heatwave’s third day, indicating a critical time window after the onset of extreme heat during which pre-hospital and hospital systems must prepare for an influx of high-acuity patients requiring substantial hospital and critical care resources. It is critical not only to activate community-based interventions, such as wellness checks and cooling centres, and ensure public awareness of simple and readily available cooling measures, but also for EMS and hospital systems to increase staffing levels and prepare equipment and physical care spaces in appropriate areas of the hospital to treat and support high-acuity heatstroke patients [33, 34]. Cooling measures were used on an ad hoc basis by EMS crews and in hospitals. While our study was non-interventional due to its nature, patients were effectively and rapidly cooled with widely available simple measures such as ice packs and wet towels. These low-tech cooling measures are inexpensive, readily available and achieved rapid cooling.

### Research implications

Future work should evaluate the effectiveness of population- and system-level interventions in extreme heat events. These might include proactive identification and maintenance of lists of older individuals who live alone in private residences who may benefit from wellness checks. Those completing wellness checks should be equipped with the simple cooling measures we observed in our study that were associated with rapid cooling. Implementation and scale-up studies may be helpful in understanding which interventions can most effectively and rapidly be deployed at scale with minimal training and at minimal cost to optimize population-level preparedness and reduce EMS activations, morbidity, hospital utilization, and deaths. Finally, interventional studies are needed to guide the prevention and treatment of heatstroke complications in.

## Conclusion

BC’s 2021 extreme heat event exemplified that “disasters occur when hazards meet vulnerability” [35]. In BC’s temperate climate with low levels of population-level preparedness, older adults living alone were highly susceptible to heatstroke, with most presenting after the third day of extreme heat. Without improving population- and system-level preparation for more frequent and severe extreme heat events, we risk repeating this pattern of vulnerability. In addition to scaling up preventative community-based interventions, EMS systems, EDs, and hospitals must prepare for an influx of high-acuity patients requiring supportive care starting 48 h into extreme heat events.

### Supplementary Information

Below is the link to the electronic supplementary material.

Supplementary file1 (DOCX 181 KB)
