# Supplementary material for: Heatstroke presentations to urban hospitals during BC’s extreme heat event: lessons for the future
Source: CJEM. 2023 Dec 28;26(2):111–8. doi: 10.1007/s43678-023-00622-y (PMC10861625; doi:10.1007/s43678-023-00622-y)
Supplement: Supplementary file 1 — Supplementary file1 (DOCX 181 KB) [file 43678_2023_622_MOESM1_ESM.docx]

**APPENDICES**

**Appendix Table 1:** Patient tracker by site

| **Sites** | **SCREENED PATIENTS n (%)** | **MET SCREENING CRITERIA** | **INCLUDED** | **INCLUDED (%)** |
| --- | --- | --- | --- | --- |
| Vancouver General Hospital | 1674 (16.3) | 695 | 52 | 3% |
| Saint Paul's Hospital | 1525 (14.9) | 584 | 12 | 1% |
| Mount Saint Joseph's Hospital | 633 (6.2) | 184 | 5 | 1% |
| Lions Gate Hospital | 1243 (12.1) | 383 | 4 | < 1% |
| Surrey Memorial Hospital | 2730 (26.6) | 1162 | 40 | 1% |
| Royal Columbian Hospital | 1483 (14.5) | 656 | 19 | 1% |
| Eagle Ridge Hospital | 959 (9.4) | 311 | 7 | 1% |
| **TOTAL** | **10247 (100)** | **3975** | **139** |  |

**Appendix Table 2:** Daily maximum and minimum temperatures over the heatwave period (June 25^th^-29^th^, 2021) compared to the 90^th^ percentile temperatures over the preceding 30 years.

| 2021 temperature | | | 90th percentile temperature over a 30-year period (1991-2020) | |
| --- | --- | --- | --- | --- |
| Date | Tmax (°C) | Tmin (°C) | Tmax (°C) | Tmin (°C) |
| 25-Jun | 25.4 | 18.1 | 24.91 | 15.52 |
| 26-Jun | 30.8 | 17.8 | 23.6 | 16.06 |
| 27-Jun | 30.5 | 20.1 | 24.55 | 15.7 |
| 28-Jun | 31.7 | 21.5 | 25.66 | 15.85 |
| 29-Jun | 32.1 | 20.4 | 26.23 | 16.26 |

**Appendix Table 3**: A list of presenting complaints chosen for screening process

| No. | Signs/Symptoms |
| --- | --- |
| 1 | Heat-related illness |
| 2 | Altered level of consciousness |
| 3 | General weakness |
| 4 | Confusion |
| 5 | Seizure |
| 6 | Shortness of Breath |
| 7 | Fever |
| 8 | Dehydration |
| 9 | Headache |
| 10 | Nausea or vomiting |
| 11 | Bizarre behaviour |
| 12 | Palpitations/irregular heart beat |
| 13 | Syncope/pre-syncope |
| 14 | Cardiac arrest (non-traumatic) |
| 15 | Chest pain |
| 16 | Vertigo |
| 17 | Gait disturbance/ataxia |
| 18 | Extremity weakness/symptoms of CVA |
| 19 | Sensory loss/Parasthesias |
| 20 | Hypoglycemia |

cva = cerebrovascular accident

**Appendix Table 4:** Interventions by Emergency Medical Services and Emergency Department

| **Interventions** | **Pre-hospital (n = 120)** | **Emergency Department**  **(n = 139)** |
| --- | --- | --- |
| **Hospital cooling** | **(%)** | |
| Any type of cooling  Ice bath  Ice pack  Cooling blankets  Wet towel  Ambient  Intravenous fluids  Total fluids given in litres (Median(IQR) | 62.5  0  43.3  < 5  15.8  5.0  25.8  1.0 (1.0 – 1.0) | 83.4  < 5^1^  42.4  25.9  18.0  3.6  67.6  1.0 (1.0 – 2.5) |
| **Average duration of cooling (in minutes)*** | **Mean ± SD** | |
| Ice pack  Cooling blankets  Wet towel  Ambient | 23.2 ± 32.4  31.0 ± 18.4  35.0 ± 29.8  32.0 ± 1.7 | 92.5 ± 76.8  60 ± 50.4  138.3 ± 132.5  NA |
| **Mechanical ventilation** | **(%)** | |
| Advanced airway placement  Endotracheal intubation  Supraglottic device  Unknown | 7.5  4.2  < 5  0 | 17.3  10.1  < 5  6.5 |
| **Medications administered** | **(%)** | |
| Vasopressors  Ketamine  Propofol  Etomidate  Rocuronium  Benzodiazepine  Antibiotics  Antiepileptics | 0  < 5  0  0  0  0  0  0 | 7.9  10.8  12.9  < 5  12.9  8.6  43.2  < 5 |

SD=standard deviation

*Average duration of cooling for patients who received ambient or ice bath in ED is not available due to missing data

^1To ensure patient privacy, a cell size restriction policy prohibited reporting counts of less than five^

**Appendix Table 5:** Health Service Utilization

| **ED Disposition, (%)** |  |
| --- | --- |
| Discharged Home  Admitted  Intensive care unit admission  Regular ward  High acuity/step down ward  Left against medical advice  Death  Other | 18.0  77.0  13.7  58.3  3.6  < 5^1^  3.6  < 5 |
| **Length of stay in ED in hours (median (IQR))** | 8.2 (1.9-14.4) |
| **Length of stay in hospital in days (median (IQR))** | 9.0 (1.0-17.0) |
| ICU (median (IQR))  Regular ward (median (IQR))  High acuity/step down ward (median (IQR)) | 3.0 (2.0-8.0)  7.0 (2.0-11.8)  8.0 (4.0-11.0) |
| **In hospital death, (%)** | 11.5 |
| Died in ED  Died on a ward, in high acuity/step down or in ICU | 3.6  7.9 |

ED=Emergency Department; ICU=intensive care unit

^1 To ensure patient privacy, a cell size restriction policy prohibited reporting counts of less than five^

**Appendix Table 6:** Comparison of laboratory data by survival status (mean ± SD))

|  | **Survivors (n= 123)** | **Non-survivors (n = 16)** | **P value** |
| --- | --- | --- | --- |
| Potassium (mmol/L)  Creatinine (μmol/L)  Alanine aminotransferase (IU/L)  Lactate (IU/L)  International normalized ratio  Troponin  pH  Venous  Arterial  pCO2  Venous  Arterial  Bicarbonate | 3.7 ± 0.6  151.4 ± 105.2  30.7 ± 28.4  2.5 ± 2.1  1.3 ± 0.4  6.0 ± 31.8  7.4 ± 0.1  7.3 ± 0.1  35.4 ± 7.6  39.5 ± 7.9  19.7 ± 3.2 | 4.4 ± 0.8  213.7 ± 103.8  114.9 ± 207.6  5.4 ± 2.0  1.8 ± 0.8  3.6 ± 6.3  7.2 ± 0.1  7.2 ± <0.1  43.4 ± 9.8  40.7 ± 3.8  18.4 ± 4.6 | < 0.01  < 0.01  0.02  < 0.01  < 0.01  < 0.01  < 0.01  0.04  0.04  0.70  0.11 |

SD = standard deviation

**Appendix Table 7:** Univariate associations between in-hospital mortality and demographic and clinical factors^1^

|  | **Survivor (n= 123)** | | **Non-survivor (n = 16)** | **Unadjusted OR (95% CI)** |
| --- | --- | --- | --- | --- |
| **Demographics** (%) | | | | |
| Age | | | | |
| 49 - 79 years  80 – 89 years  90+ years | | 33.3  39.8  26.8 | < 5^4^  43.8  37.5 | Reference  1.95 (0.51, 9.50)  2.48 (0.61, 12.48) |
| Sex | | | | |
| Male  Female | 44.7  55.3 | | 43.8  56.2 | Reference  1.03 (0.36, 3.07) |
| EMS Caller^2^ | | | | |
| Anyone other than self  Self/Unclear | 83.0  17.0 | | 81.3  < 5 | Reference  0.38 (0.02, 2.08) |
| Time to the ED arrival (in hour)^3^ | | | | 0.46 (0.10, 1.02) |
| **Comorbidities** (%) | | | | |
| Diabetes mellitus | | | | |
| No  Yes | 66.7  33.3 | | 75  < 5 | Reference  0.67 (0.18, 2.05) |
| Hypertension | | | | |
| No  Yes | 43.1  56.9 | | 43.8  56.2 | Reference  0.97 (0.34, 2.89) |
| Congestive heart failure | | | | |
| No  Yes | 81.3  18.7 | | 68.8  31.2 | Reference  1.98 (0.58, 6.02) |
| Psychiatric condition/mental health diagnosis | | | | |
| No  Yes | 71.5  28.4 | | 87.5  < 5 | Reference  0.36 (0.05, 1.37) |
| Chronic kidney disease | | | | |
| No  Yes | 86.2  13.8 | | 87.5  < 5 | Reference  0.89 (0.13, 3.58) |
| **Vital Sign** (%) | | | | |
| Respiratory rate, breaths per min | | |  | 1.00 (0.99, 1.01) |
| Tachycardia (HR >=100) | | | | |
| No  Yes | 22.8  77.2 | | 43.8  56.2 | Reference  0.38 (0.13, 1.14) |
| Hypotension (SBP <100) | | | | |
| No  Yes | 80.5  19.5 | | 43.8  56.2 | Reference  5.30 (1.80, 16.3) |
| Time to normothermia (<38°C, in hours) | | | | 0.99 (0.19, 1.63) |

CI = confidence interval; ED = Emergency Department; EMS = Emergency Medical Service

^1^ Due to the small sample size, this estimate needs to be interpreted with caution.

^2^ 120 patients arrived by Emergency Medical Service

^3^The time from EMS call initiated to ED arrival

^4 To ensure patient privacy, a cell size restriction policy prohibited reporting counts of less than five^

**Appendix Figure 1:** Change in patient body temperature from first contact by EMS or ED arrival over time with LOESS line and 95% confident intervals


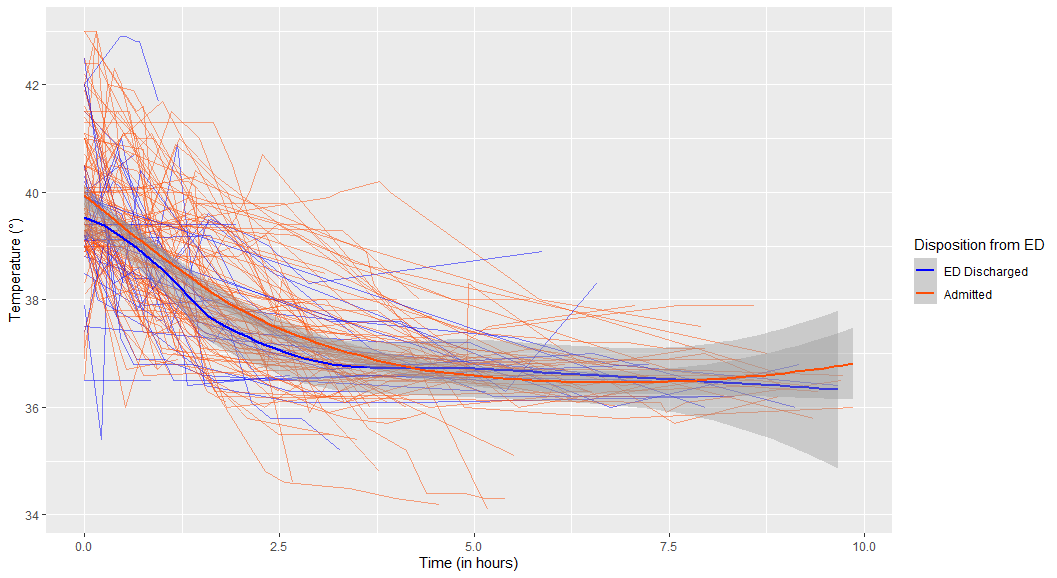


EMS = Emergency Medical Service; ED = Emergency Department; LOESS = locally estimated scatterplot smoothing

**Appendix Figure 2**: a) Heatstroke presentations to participating EDs between June 25, 2021, and June 30, 2021 with minimum and maximum temperatures and b) proportion of ED patient presentations following heatwave onset, starting from midnight June 25, 2021.

(a)


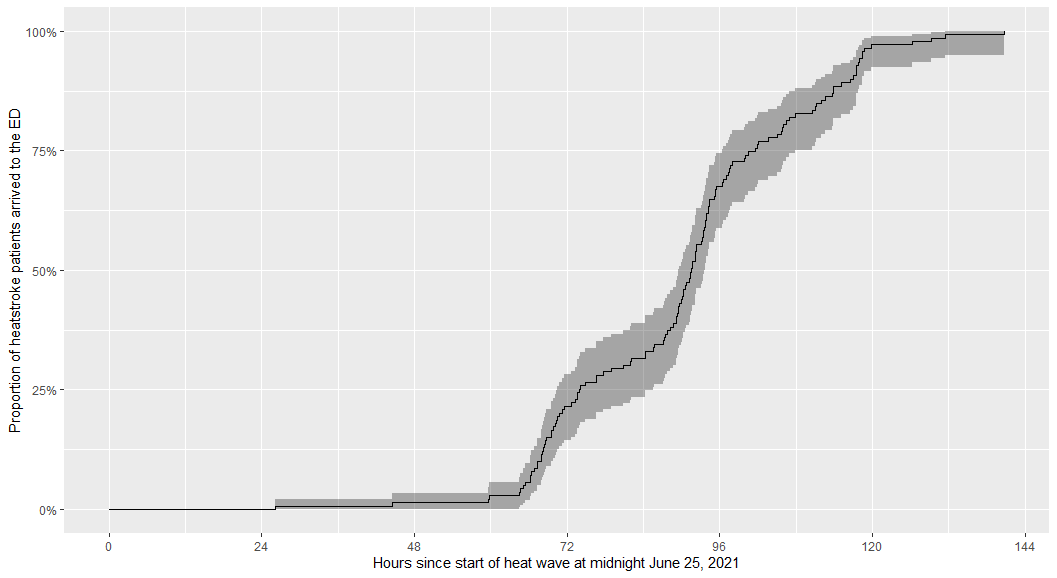


(b)

ED = Emergency Department
